# Impact of Treatment on Rate of Biphasic Reaction in Children with Anaphylaxis

**DOI:** 10.5811/westjem.18555

**Published:** 2024-10-29

**Authors:** William Bonadio, Connor Welsh, Brad Pradarelli, Yunfai Ng

**Affiliations:** Mount Sinai Morningside Medical Center, New York, New York

## Abstract

**Objective:**

Our goal was to characterize a large group of children presenting to the emergency department (ED) with acute anaphylaxis, treated with intramuscular epinephrine (IM EPI) and a corticosteroid (CS), and to determine the impact of pharmacologic intervention on the rate and timing of biphasic reactions (BPR).

**Methods:**

We reviewed consecutive children diagnosed with acute anaphylaxis managed in three EDs during a six-year period. All received IM EPI and CS, followed by monitoring for 4–6 hours post-treatment. We analyzed the rate and timing of BPR, comparing the intervals of 0–4 vs 4–48 hours after initiating therapy.

**Results:**

During the study period, there were 371 cases of anaphylaxis, of which 357 (94%) received both IM EPI and CS. Of these, 49 (14%) manifested BPR [84% had received prehospital IM EPI] requiring at least one additional dose of IM EPI [14% required ≥2 additional doses]. All BPR episodes occurred within the 0–4 hour interval after initiating therapy, whereas no patient manifested a BPR requiring an additional dose of IM EPI during the 4–48 hours after initiating therapy (*P* = <0.001, 95% CI 0–1.3%). No patient returned to the ED with recurrence of anaphylaxis symptoms within 48 hours after discharge.

**Conclusion:**

Approximately 1 in 7 children with anaphylaxis experience a biphasic reaction after receiving intramuscular epinephrine. Children with anaphylaxis who exhibit symptomatic resolution four hours following initiation of therapy have a low risk for subsequently developing BPR. Most BPR cases required only one additional dose of IM EPI to effect resolution. The rate of BPR in those receiving IM EPI and a corticosteroid is significantly lower >4 hours vs <4 hours after initiating therapy.

Population Health Research Capsule What do we already know about this issue?

*The optimal interval of ED observation for treated children with anaphylaxis, and the impact of corticosteroids in modifying risk for biphasic reaction (BPR), are unknown.*

What was the research question?

*How does epinephrine and corticosteroid therapy impact the rate and timing of BPR?*

What was the major finding of the study?

*All BPR episodes occurred <4 hrs after initiating therapy; no BPR case required parenteral epinephrine during 4–48 hrs after initiating therapy (P < 0.001, 95% CI 0–1.3%).*

How does this improve population health?

*These results help to define optimal monitoring period for treated children with anaphylaxis and shed light on corticosteroid impact on BPR recurrence rate.*

## INTRODUCTION

Anaphylaxis is a common and potentially severe, even lethal, systemic allergic reaction that is increasing in frequency.^1^ In those patients who experience this condition and clinically improve after receiving medication, there is potential for a biphasic reaction (BPR) to manifest as symptomatic recurrence/exacerbation. Prior research has documented rates of BPR associated with anaphylaxis widely ranging from 1–23%.^1^

Some have recommended against routinely treating acute anaphylaxis with corticosteroids (CS) due to lack of proven efficacy.^2^ ^,^ ^3^ Yet it is plausible that the anti-inflammatory qualities of this medication could potentially ameliorate the IgE-mediated anaphylaxis process.^4^ ^,^ ^5^ Since the effect of intramuscular epinephrine (IM EPI) is short-lived, lasting briefly (t½ of 2–3 minutes), and CS has clinical onset approximately >4 hours following administration, lasting beyond 24 hours, it is likely that after >4 hours the IM EPI effect wanes and any ongoing anti-allergy effect is likely due to CS. The BPR has been shown to potentially manifest at any time during the subsequent 48 hours after onset (median time 18.5 hours in one study^6^); so, it should be therapeutically advantageous to administer a longer acting anti-allergy medication like CS to help prevent this complication.

The purpose of this study was to analyze a large number of pediatric cases of anaphylaxis treated with IM EPI and CS to determine whether a correlation exists between pharmacologic intervention and the rate and timing of BPR.

## METHODS

We conducted a retrospective cohort study of consecutive children aged <19 years managed in three EDs at the Mount Sinai Medical Center in New York between January 2015–December 2022, inclusive; two are dedicated pediatric EDs, and one is a general ED also managing children (combined yearly pediatric census 55,000 child-visits). Our templated electronic health record (EHR) (Epic Systems Corporation Verona, WI) stereotypically queries an extensive roster of potential anaphylaxis symptoms, involving dermal, cardiovascular, ear nose throat, pulmonary, neurologic, and gastrointestinal systems. The EHR also documents subsequent medical visits throughout other area hospitals/clinics. Anaphylaxis was diagnosed, per previously published criteria,^7^ with acute onset of multiple symptoms following exposure to allergen trigger involving at least two organ systems (Table 1).

**Table 1.** Symptoms of anaphylaxis.

• Skin/mucosa: urticaria, pruritis, flushing, facial/mucosal angioedema
• Respiratory: wheezing, cough, stridor, dyspnea
• Cardiovascular: tachycardia, hypotension
• Gastrointestinal: nausea, vomiting, diarrhea, abdominal pain
• Oropharynx: throat swelling, oral pruritis

We defined BPR as a recurrence/exacerbation of symptoms following administration of IM EPI, after a period of partial or complete recovery, without re-exposure to the trigger, indicative of anaphylaxis or generalized allergic reaction of sufficient severity to require at least one additional dose of epinephrine.

The ED management of anaphylaxis was nearly unanimous among all clinicians, consisting of IM EPI and CS in 96% of cases. The CS medications included dexamethasone, methylprednisolone, prednisolone, or prednisone (intravenously or orally) on the day of presentation. All patients with anaphylaxis received ED monitoring for 4–6 hours post-therapy initiation for BPR. Our templated discharge instructions for “anaphylaxis” states patients should seek prompt medical attention if any of the following occur: 1) worsening of symptoms; 2) trouble breathing or swallowing; 3) swelling of mouth or face; 4) chest pain; and 5) dizziness, weakness or fainting.

We also surveyed the subsequent EHR documentation to determine whether a repeat visit to an ED or other outpatient facility occurred within 48 hours of discharge. In patients with anaphylaxis who received both IM EPI and CS, the primary outcome measure was to determine 1) the rate of BPR, and 2) the comparative rates of BPR in 0–4 and 4–48 hours post receiving IM EPI. The secondary outcome measure was to determine the rate of ED return visit for BPR within 48 hours of ED discharge.

### Statistical Considerations

Categorical data are described in terms of frequency (%) and compared using *κ* coefficients. We calculated a sample of 280 cases to allow for 80% power (alpha 0.05) to determine at least a 10% difference in BPR rates between intervals of <4 hours (15%) vs >4 hours (5%) after CS administration.^1^ ^,^ ^8^ To determine inter-rater agreement in collating results, 30 cases (15% of total) were randomly selected and eight variables compared between reviewers; the kappa was 0.84, indicative of substantial agreement. Since there were fewer than five patients in one cell, we performed a Fisher exact test comparing rates of BPR during the 0–4 vs >4 hours post-treatment intervals. We calculated binomial probability 95% confidence interval (CI) for those who received IM EPI and CS with no BPR occurrence, using the Clopper-Pearson exact method (CI 77–84%). The study was approved by the Icahn School of Medicine Investigational Review Board.

## RESULTS

During the study period, there were 371 consecutive cases of anaphylaxis involving 280 patients; in 357 cases (94%) both IM EPI and CS were administered. Each record in the study cohort had a completed attending-level physician note reviewing history of present illness, review of symptoms, physical exam, and medical decision-making. The demographic profile of this cohort is given in Table 2. A total of 49 patients (14%) manifested BPR requiring at least one additional dose of IM EPI [14% required ≥2 additional doses]; 41 [84%] had received prehospital administration of IM EPI. All BPR events occurred during the initial four hours after initiating therapy, whereas no patient manifested BPR requiring an additional dose of IM EPI during the latter 4–48 hour interval after initiating therapy (*P* = <0.001). No patient was documented to return to the ED with recurrence or exacerbation of anaphylaxis symptoms within 48 hours after discharge.

**Table 2.** Patient characteristics: 357 cases of anaphylaxis treated with intramuscular epinephrine and corticosteroid.

Variable	N [%]
Patient age range	3 months–19 years
Patient age median	9.2 years
Patient sex	
• Male	181 [50.7%]
• Female	176 [49.3%]
ED length of stay (median)	4.7 hours
ED length of stay (range)	3.5–6.2 hours
Anaphylaxis trigger	
• Food exposure	328 [93%]
• Unknown	20 [5.0%]
• Environmental exposure	4 [1.0%]
• Drug-related	5 [1.0%]
Management received – all cases:	
• IM EPI	357 [100%]
• Prehospital IM EPI	193 [54%]
• CS	357 [100%]
• H1 and/or H2 receptor antagonist*	344 [96%]
Biphasic reaction cases^	
• Total	49 [14%]
• received >1 dose of IM EPI	49 [14%]
• received >2 doses of IM EPI	8+ [2%]
• received prehospital IM EPI	41 [84%]
• during the initial 4 hours after initiating therapy	49 [100%]#
• during the interval 4–48 hours after initiating therapy	0#
Inpatient hospitalization	11 [3%]
Deaths	0

* Receptor antagonist H1 = diphenhydramine; H2 = famotidine.

^ Within 48 hours after initial presentation.

+ All doses of IM EPI were given during the initial 0–4 hours after initiating ED therapy.

# *P*-value comparing rates is *P* < 0.001.

*BPR*, biphasic reaction; *CS*, corticosteroid; *ED*, emergency department; *IM EPI*, intramuscular epinephrine.

## DISCUSSION

In our analysis we sought to review a large number of pediatric anaphylaxis cases treated with IM EPI and CS to determine the subsequent rate and timing of BPR. Our approach to management was nearly unanimous, as 96% of cases received IM EPI and CS. The 14% observed rate of BPR, and predominance of food allergen triggers, are consistent with prior pediatric reports.^9^ ^,^ ^10^ The majority (84%) of BPR cases required only one additional dose of IM EPI to effect resolution.

Using our institutional approach, the 0% BPR rate achieved during the 4–48 hour interval after initiating therapy is lower than has been previously reported in the literature; several of those analyses found no significant difference in return rates between those who did vs those who did not receive CS.^11^ ^,^ ^12^ The factor of time delay from entry into medical care to initial dose of IM EPI impacting BPR rate was minimized in our cohort, as 84% of patients developing this complication had received rapid deployment of an initial dose of IM EPI in the prehospital phase of management. Recent review articles on pediatric anaphylaxis recommend against CS treatment, due to lack of proven efficacy.^2^ ^,^ ^3^ One^13^ implicated CS treatment as increasing “the likelihood of a biphasic reaction in children by as much as 50%” (no study citation given). A foreign retrospective study surveying a large database comparing anaphylaxis treatment with and without CS found no significant difference in BPR rates yet did not specifically correlate onset of BPR with timing of CS administration.^14^

The exact pathogenesis of anaphylactic BPR is unclear; potential theories include a second wave of mast cell degranulation, delayed ongoing absorption of offending antigen (especially oral antigenic exposures), or the waning effect of therapy.^15^ As an extension of treatment for other atopic conditions, CS therapy is expected to exert a beneficial effect in modulating the IgE-mediated process of anaphylaxis. This class of drugs has proven efficacy in ameliorating other allergic-mediated conditions such as urticaria and asthma. There is evidence that CS can inhibit mast cells, which are strongly implicated in the anaphylaxis cascade, by down-regulating pro-inflammatory cytokine transcription, regulating multiple adaptor and signaling molecules, and rapidly decreasing cell histamine release.^4^ ^,^ ^5^ These characteristics support the biological plausibility of CS affecting BPR prevention. Consistent with this, a prior study^12^ of pediatric anaphylaxis showed early CS therapy was inversely associated with prolonged length of stay and subsequent IM EPI requirement among those hospitalized.

There are no published prospective randomized, placebo-controlled studies assessing CS effect in treating anaphylaxis. While such studies and their data would be an important step toward a more complete understanding of BPR in anaphylaxis, the design of such a study would be problematic. Obtaining informed consent to randomize treatment would be prohibitive for the potentially lethal medical condition of acute anaphylaxis, in which delay in therapeutic intervention measured in minutes can be crucial in determining outcome. Due to ethical considerations, such a study would mandate treatment of all patients with IM EPI and then compare a group treated with CS vs placebo. The intervals of analysis compared would be prior to vs after CS therapeutic onset, likely at the four-hour post-administration mark. Our methodology largely simulates this scheme; yet any analysis including IM EPI treatment of all patients still leaves unanswered the question of whether the initial IM EPI dose actually “resolves” anaphylaxis vs merely providing a temporizing suppression of the reaction—with CS exerting a further effect in BPR prevention.

Our findings may shed light on a potential CS therapeutic impact on rate of BPR recurrence. Since in all instances both IM EPI and CS were administered, we noted a significant difference in BPR rates when partitioning clinical course based on medication pharmacokinetics. The IM EPI effect is rapid in onset, lasting briefly (t½ of 2–3 minutes), whereas CS has clinical onset approximately >4 hours following administration, lasting beyond 24 hours.^16^ ^–^ ^18^ It is likely that after four hours the IM EPI effect wanes, and any ongoing anti-allergy effect is likely due to CS. Within this context, we found the rate of BPR requiring repeat IM EPI administration was significantly lower 4–48 hours vs 0–4 hours after initiating therapy.

## LIMITATIONS

As with any observational study, data gathering was limited by information present. We largely avoided this deficiency, in that nearly all our practitioners managed patients and thoroughly documented management in a stereotyped manner. We did not have sufficient data to analyze time from onset of anaphylaxis symptoms to receiving IM EPI and its potential impact on BPR rate.

## Conclusion

Approximately 1 in 7 children with anaphylaxis experience a biphasic reaction after receiving intramuscular epinephrine. Children with anaphylaxis who exhibit symptomatic resolution 4–6 hours following initiation of therapy have a low risk for subsequently developing BPR. The majority of BPR cases require one additional dose of IM EPI to effect resolution. The rate of BPR in those receiving IM EPI and corticosteroids is significantly lower 4–48 hours vs 0–4 hours after initiating therapy.

## References

[1] Patek P Owda D Menoch M . Anaphyl-crisis: rising rates of pediatric anaphylaxis. Pediatr Emerg Care 2022;38(9):e1529–32. 35639391 10.1097/PEC.0000000000002771

[2] Alqurashi W Ellis AK . Do corticosteroids prevent biphasic anaphylaxis? Allergy Clin Immunol Pract 2017;5(5):1194–1205. 10.1016/j.jaip.2017.05.022 28888249

[3] Choo KJL Simons E Sheikh A . Glucocorticoids for the treatment of anaphylaxis: Cochrane systematic review. Allergy 2010;65(10):1205–11. 20584003 10.1111/j.1398-9995.2010.02424.x

[4] Caslin HL Kiwanuka KN Haque TT et al . Controlling mast cell activation and homeostasis: work influenced by Bill Paul that continues today. Front Immunol 2018;9:868. 29755466 10.3389/fimmu.2018.00868 PMC5932183

[5] Oppong E Flink N Cato ACB . Molecular mechanisms of glucocorticoid action in mast cells. Mol Cell Endocrinol 2013;380:119–26. 23707629 10.1016/j.mce.2013.05.014

[6] Alqurashi W Stiell I Chan K et al . Epidemiology and clinical predictors of biphasic reactions in children with anaphylaxis. Ann Allergy Asthma Immunol 2015;115:217–23.e2. 26112147 10.1016/j.anai.2015.05.013

[7] Cardona V Ansotegui IJ Ebisawa M et al . World Allergy Organization anaphylaxis guidance 2020. World Allergy Organ J 2020;30;13(10):100472. 33204386 10.1016/j.waojou.2020.100472 PMC7607509

[8] Tarczoń I Cichocka-Jarosz E Knapp A et al . The 2020 update on anaphylaxis in pediatric population. Postepy Dermatol Alergol 2022;39(1):13–9. 35369644 10.5114/ada.2021.103327 PMC8953896

[9] Sampson HA Muñoz-Furlong A Campbell RL et al . Second symposium on the definition and management of anaphylaxis: summary report—Second National Institute of Allergy and Infectious Disease/Food Allergy and Anaphylaxis Network Symposium. J Allergy Clin Immunol 2006;117(2):391–7. 16461139 10.1016/j.jaci.2005.12.1303

[10] Tejedor A Moro M García M . Epidemiology of anaphylaxis. Clin Exp Allergy 2015;45:1027–39. 25495512 10.1111/cea.12418

[11] Liyange CK Galappathy P Senevir SL . Corticosteroids in management of anaphylaxis; a systematic review of evidence. Eur Ann Allergy Clin Immunol 2017;49(5):196–207. 28884986 10.23822/EurAnnACI.1764-1489.15

[12] Michelson KA Monuteaux MC Neuman MI . Glucocorticoids and hospital length of stay for children with anaphylaxis: a retrospective study. J Pediatr 2015;167(3):719–24.e1–3. 26095285 10.1016/j.jpeds.2015.05.033

[13] Tanverdi MS Wiersma A Kim KM et al . Anaphylaxis in children. Pediatr Emerg Care 2022;38:456–61. 36040466 10.1097/PEC.0000000000002812

[14] Nagata S Ohbe H Jo T et al . Glucocorticoids and rates of biphasic reactions in patients with adrenaline-treated anaphylaxis: a propensity score matching analysis. Int Arch Allergy Immunol 2022;183(9):939–45. 35526528 10.1159/000524612 PMC9533429

[15] Lee S Bellolio MF Hess EP et al . Time of onset and predictors of biphasic anaphylactic reactions: a systematic review and meta-analysis. J Allergy Clin Immunol Pract. 2015;3(3):408–16. 25680923 10.1016/j.jaip.2014.12.010

[16] Samuel S Nguyen T Choi A . Pharmacologic characteristics of corticosteroids. J Neurocrit Care 2017;10:53–9.

[17] Dreborg S Kim H . The pharmacokinetics of epinephrine/adrenaline auto-injectors. Allergy Asthma Clin Immunol. 2021;25.33685510

[18] Shaker M Wallace D Golden D et al . Anaphylaxis: a 2020 practice parameter update, systematic review, and grading of recommendations, assessment, development and evaluation (GRADE) analysis. J Allergy Clin Immunol. 2020;145(4):1082–123. 32001253 10.1016/j.jaci.2020.01.017

